# Proanthocyanidins-Based Synbiotics as a Novel Strategy for Nonalcoholic Fatty Liver Disease (NAFLD) Risk Reduction

**DOI:** 10.3390/molecules29030709

**Published:** 2024-02-03

**Authors:** Wasitha P. D. W. Thilakarathna, H. P. Vasantha Rupasinghe

**Affiliations:** 1 Department of Plant, Food, and Environmental Sciences, Faculty of Agriculture, Dalhousie University, Truro, NS B2N 5E3, Canada; wasitha@dal.ca; 2 Department of Pathology, Faculty of Medicine, Dalhousie University, Halifax, NS B3H 4H7, Canada

**Keywords:** nonalcoholic fatty liver disease, steatosis, nonalcoholic steatohepatitis, synbiotics, proanthocyanidins, flavonoids, probiotic bacteria

## Abstract

Nonalcoholic fatty liver disease (NAFLD), the most common liver disease worldwide, is a spectrum of liver abnormalities ranging from steatosis to nonalcoholic steatohepatitis (NASH) characterized by excessive lipid accumulation. The prevalence of NAFLD is predicted to increase rapidly, demanding novel approaches to reduce the global NAFLD burden. Flavonoids, the most abundant dietary polyphenols, can reduce the risk of NAFLD. The majority of dietary flavonoids are proanthocyanidins (PACs), which are oligomers and polymers of the flavonoid sub-group flavan-3-ols. The efficacy of PAC in reducing the NAFLD risk can be significantly hindered by low bioavailability. The development of synbiotics by combining PAC with probiotics may increase effectiveness against NAFLD by biotransforming PAC into bioavailable metabolites. PAC and probiotic bacteria are capable of mitigating steatosis primarily through suppressing de novo lipogenesis and promoting fatty acid *β*-oxidation. PAC and probiotic bacteria can reduce the progression of steatosis to NASH mainly through ameliorating hepatic damage and inflammation induced by hepatic oxidative stress, endoplasmic reticulum stress, and gut microbiota dysbiosis. Synbiotics of PAC are superior in reducing the risk of NAFLD compared to independent administration of PAC and probiotics. The development of PAC-based synbiotics can be a novel strategy to mitigate the increasing incidence of NAFLD.

## 1. Introduction

Nonalcoholic fatty liver disease (NAFLD) is the primary cause of chronic liver diseases in the world [1]. About 30% of the global population is affected by NAFLD [2], and the global prevalence of NAFLD among the adult population is predicted to increase up to 55.7% by 2040 [3]. The term NAFLD is collectively used to describe hepatic abnormalities characterized by excessive lipid accumulation in the absence of alcohol consumption at levels harmful to the liver. These abnormalities can vary from simple steatosis to nonalcoholic steatohepatitis (NASH), which may progress to fibrosis, cirrhosis, and hepatocellular carcinoma (HCC) [4]. Steatosis, also known as nonalcoholic fatty liver, is defined as the presence of intrahepatic lipids over 5% of the liver weight or the presence of lipid vacuoles in more than 5% of the hepatocytes [4,5]. Simple steatosis can progress into NASH in 20–30% of NAFLD patients with the occurrence of lobular inflammation and hepatocyte ballooning [6]. Chronic liver damage by steatosis and NASH can lead to fibrosis by creating fibrous scars. These fibrous scars can alter hepatic parenchyma structure and, together with nodules developed by the regenerating hepatocytes, can be manifested as hepatic cirrhosis [7]. Chronic liver injury and cirrhosis are the primary elicit of HCC. In fact, 80% of HCC cases onset under the presence of cirrhosis and chronic liver diseases [8].

Obesity is a primary risk factor for NAFLD development. The increased prevalence of NAFLD is directly proportional to the increasing prevalence of obesity [9]. Obesity is a major risk factor for insulin resistance, type-2 diabetes (T2D), and metabolic syndrome (MetS). These conditions can induce the pathogenesis of NAFLD and commonly coexist in NAFLD patients [10]. Smoking is another modifiable lifestyle risk factor of NAFLD. The NAFLD risk is considerably high among current, past, and passive smokers [11]. Apart from the risk factors linked with poor lifestyle choices, age, gender, ethnicity, and genetic makeup can predispose some individuals to NAFLD. The risk of NAFLD considerably increases with age [12], and women are more resilient to NAFLD owing to the estrogen-mediated protection against dysmetabolism and inflammation [13]. The variations in food patterns, lifestyle, and socioeconomic status, together with genetics, may create the NAFLD prevalence heterogeneity between different ethnic populations [14].

Currently, a drug approved by the United States Food and Drug Administration (FDA) or the European Medical Agency is not available to treat NAFLD [15]. The newly emerging therapeutic approaches target the root causes of NAFLD, especially insulin resistance and T2D. Glucagon-like peptide (GLP)-1 modulators, thiazolidinedione insulin sensitizers, and sodium-glucose cotransporter (SGLT)-2 inhibitors are among the most promising antidiabetic agents to treat NAFLD [16]. Also, two experimental drugs, Obeticholic acid and resmitirom, are currently being evaluated by the FDA as potential treatments for NAFLD [17]. The increasing prevalence, together with the unavailability of an approved drug, demands alternative approaches to mitigate the risk of NAFLD. Recent studies have suggested that dietary polyphenols can be highly effective for the reduction of NAFLD risk [18]. There are more than 25,000 natural phenolic compounds, making polyphenols one of the largest groups of phytochemicals [19]. Polyphenols can be classified as phenolic acids, flavonoids, stilbenes, and lignans due to their extensive structural diversity [20]. Flavonoids are the most abundant polyphenols in the human diet [21,22], and the majority of dietary flavonoids are proanthocyanidins (PACs) [23]. PACs, also known as condensed tannins, are oligomeric and polymeric molecules resulting from the condensation of flavan-3-ols (mostly catechin and epicatechin), a sub-group of flavonoids [24]. The differentiation of oligomeric PAC from polymeric PAC is based on molecular size. PAC molecules with 2–10 monomers are the oligomers, and PACs larger than 10 monomers are the polymers [25]. PACs are ubiquitously found in plant-based food, including berries, nuts, cereals, beans, and their products [26]. PAC can alleviate NAFLD through multiple mechanisms of cellular lipid metabolism and inflammation, together with the mitigation of primary risk factors, including obesity, T2D, and MetS [27]. However, the therapeutic efficacy of PAC in vivo can be considerably low due to low bioavailability. The majority of ingested PACs evade absorption in the small intestine and reach the colon. In a human study, 70% of the ingested green tea flavanols had reached the ileum without being absorbed [28]. The colonic microbiota can degrade the unabsorbed PAC into simple metabolites, mainly phenolic acids and *γ*-valerolactones [29]. However, the colonic catabolism of PAC is inefficient [30], demanding efficient methods to degrade PAC into bioactive and bioavailable metabolites. Probiotic bacteria can biotransform PAC into simple metabolites [31], and bioactivities of such metabolites have already been established in disease models [32]. Thus, the development of PAC-based synbiotics can be beneficial to reduce the burden of NAFLD worldwide. Synbiotics are the health-beneficial combinations of probiotics together with substrates (prebiotics) selectively utilized by the probiotic bacteria [33]. In this review, we discuss the pathogenesis of steatosis and its progression to NASH by the overexpression of fatty acid (FA) transporters, disruption of FA oxidation and hepatic triglyceride (TG) secretion, hepatic oxidative and endoplasmic reticulum (ER) stresses, adipose tissue dysfunction, and gut microbiota dysbiosis. We also comprehensively explore the possibility of utilizing probiotics and PAC together with the potential to develop PAC-based synbiotics to reduce the risk of NAFLD.

## 2. Pathogenesis of Steatosis and NASH

**Figure 1:**
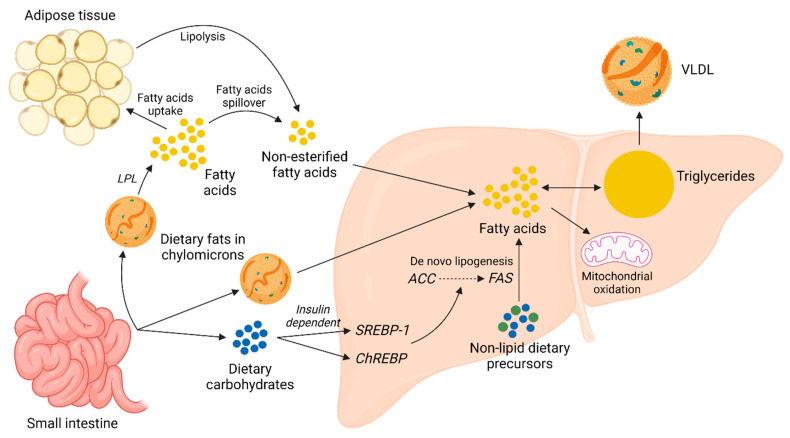
Hepatic lipid metabolism. ACC, acetyl-CoA carboxylase; ChREBP, carbohydrate response element-binding protein; FAS, fatty acid synthase; LPL, lipoprotein lipase; SREBP-1, sterol regulatory element binding protein-1; VLDL, very-low-density lipoprotein (created with BioRender.com, a license purchased, accessed on 14 November 2023).

The pathogenesis of steatosis occurs due to the imbalance between hepatic TG accumulation and expenditure. The liver is not the primary location of TG storage, yet it is the central organ for lipid metabolism [34]. The TG in the liver can derive from the diet, de novo lipogenesis (DNL), and non-esterified FA/free fatty acids (FFA). The serum FFA can originate from adipose tissue lipolysis and FA spillover during chylomicron degradation (Figure 1). In NAFLD patients, the contribution of diet, DNL, and FFA to hepatic TG accumulation is 14.9%, 26.1%, and 59%, respectively [35]. Therefore, diet, DNL, and FFA are therapeutic targets with similar importance for steatosis mitigation. Postprandial lipid metabolism is a finely regulated complex process. Briefly, dietary FA absorbed in the gut is converted to TG in the enterocytes and temporarily stored as cytoplasmic lipid droplets or assembled into chylomicrons [36]. These chylomicrons enter the bloodstream through the lymphatic system. The adipose tissues and skeletal muscles can uptake the TG in chylomicrons by the activity of the lipoprotein lipase (LPL). The remnants of chylomicrons that remain after unloading the TG content are cleared by the liver. However, under an excessive abundance of dietary lipids, the adipose and muscle tissues fail to uptake all the FA released from chylomicrons, creating a spillover of FFA [37]. Excessive dietary carbohydrate (sugars) intake can create hyperglycemia and hyperinsulinemia conditions that upregulate carbohydrate response element-binding protein (ChREBP) and sterol regulatory element-binding protein (SREBP)-1, respectively. Both ChREBP and SREBP-1 can promote DNL by the upregulation of acetyl-CoA carboxylase (ACC) and fatty acid synthase (FAS) [38]. ACC is the rate-limiting enzyme of FA synthesis. The liver attempts to prevent excessive lipid accumulation by increasing TG secretion in very low-density lipoprotein (VLDL) and promoting FA *β*-oxidation in mitochondria [37].

### 2.1. Overexpression of FA Transporters

**Figure 2:**
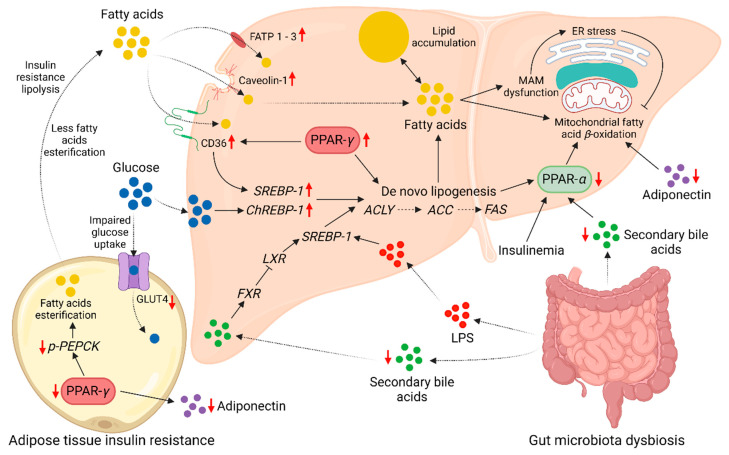
Mechanisms of steatosis pathogenesis. The red arrows indicate the increased or decreased expression/production under hepatic steatosis. ACC, acetyl-CoA carboxylase; ACLY, adenosine triphosphate citrate lyase; CD36, cluster of differentiation 36; ChREBP, carbohydrate response element-binding protein; ER, endoplasmic reticulum; FAS, fatty acid synthase; FATP, fatty acid transport protein; FXR, farnesoid X receptor; GLUT4, glucose transporter type 4; LPS, lipopolysaccharide; LXR, liver X receptor; MAM, mitochondria-associated ER membrane; PPAR, peroxisome proliferator-activated receptor; *p*-PEPCK, phosphorylated phosphoenolpyruvate carboxylase; SREBP-1, sterol regulatory element binding protein-1 (created with BioRender.com, a license purchased, accessed on 14 November 2023).

During the pathogenesis of steatosis, substantial alterations in the hepatic lipid metabolism can arise to favor excessive lipid accumulation (Figure 2). There are multiple membrane proteins in the hepatocytes to facilitate FFA uptake from the bloodstream. FA transport proteins (FATP), cluster of differentiation 36 (CD36)/fatty acid translocase, and caveolin-1 are the major proteins involved in the transmembrane influx of FAs [39]. Hepatic expression of the FATP can significantly increase during the pathogenesis of NAFLD. Overexpression of FATP1, FATP2, and FATP3 has been confirmed at the hepatic mRNA level in a high-fat diet (HFD)-fed mouse model [40]. CD36 is another well-known FFA transporter overexpressed in NAFLD. Increased expression of CD36 had been observed at hepatic mRNA and protein levels in both steatosis and NASH patients. Interestingly, overexpression of CD36 can be driven by multiple risk factors of NAFLD, including insulin resistance, hyperinsulinemia, and hepatitis C virus (HCV) infection [41]. The role of CD36 in NAFLD pathogenesis is not merely limited to FFA uptake. CD36 can activate insulin-dependent DNL through the SREBP-1-mediated activation of the adenosine triphosphate (ATP) citrate lyase (ACLY), ACC, and FAS lipogenic enzymes. CD36 promotes the activation of SREBP-1 by binding with the insulin-induced gene-2 (INSIG2). The unavailability of INSIG2 to bind with SREBP cleavage-activating protein (SCAP) facilitates the translocation of SREBP-1 from the ER to Golgi for processing and subsequent activation [42]. The ability of CD36 to disrupt FA *β*-oxidation can further promote hepatic lipid accumulation. Palmitoylation of CD36 restricts FA *β*-oxidation in HepG2 cells in vitro. Inhibition of CD36 palmitoylation can re-establish FA *β*-oxidation by activating the 5′ adenosine monophosphate-activated protein kinase (AMPK) pathway [43]. Also, inhibition of palmitoylation promotes the translocation of CD36 onto the mitochondrial membrane. On the mitochondrial membrane, CD36 acts as a bridge to transfer long-chain FA (LCFA) to long-chain acyl-CoA synthase 1 (ACSL1) to produce acyl-CoA to undergo *β*-oxidation [44]. The overexpression of CD36 is associated with the progression of hepatic steatosis to NASH by the induction of hepatic inflammation. There is a positive correlation between CD36 expression and hepatocyte apoptosis in obese NASH patients. Hepatocyte apoptosis can trigger inflammation and fibrosis of the liver tissue [45]. Moreover, palmitoylated CD36 may induce hepatic inflammation through the c-Jun-N-terminal kinase (JNK) and nuclear factor-kappa-light-chain-enhancer of activated B cells (NF-*κ*B) signaling [43]. The ability of CD36 to identify pathogen-associated molecular patterns (PAMP) and damage-associated molecular patterns (DAMP) can promote hepatic inflammation by the activation of mitogen-activated protein kinases (MAPK) and NF-*κ*B inflammatory cascades [46].

Cellular FA uptake can also occur through the special invaginations of the cell membrane known as caveolae lipid rafts. Caveolin-1 is an FA binding protein of the caveolae lipid rafts [47]. A significant hepatic upregulation of the expression of caveolin-1 had been observed in mice with NAFLD [48]. Moreover, caveolin-1 may regulate cellular FA uptake by controlling the availability of CD36 in the cell membrane [49]. Similar to CD36, caveolin-1 may also have multiple roles in the progression of NAFLD. Caveolin-1 is overexpressed in HCC patients with a history of NAFLD. Overexpression of caveolin-1 can increase the proliferation and ensure the survival of the hepatoma cells in vitro [50]. However, a recent study contradicts these findings and suggests a beneficial role of caveolin-1 in the mitigation of NAFLD. In this study, a significant reduction of the hepatic caveolin-1 levels had been observed in mice fed with an HFD. Restoration of the depleted caveolin-1 levels by using caveolin-1 scaffolding domain peptides mitigated the hepatic lipid accumulation and promoted autophagy in the mice [51]. The transmembrane FA transporters play a key role in NAFLD pathogenesis by acting as gateways for excessive FFA influx and regulation of hepatic lipid metabolism and inflammation.

### 2.2. Disruption of FA Oxidation

The hepatocytes attempt to regulate the excessive influx of FFA by *β*-oxidation in mitochondria and excretion in VLDL [37]. The catabolism of FA is dependent on the transport of FA into the mitochondrial matrix to undergo *β*-oxidation [52]. Initially, the LCFA is converted into acyl-CoA and esterified with the L-carnitine to produce acetyl-carnitine. Esterification of the acyl-CoA with L-carnitine is facilitated by the carnitine-palmitoyl-transferase-1 (CPT1) or carnitine-acyl-transferase-1 (CAT1) located on the outer membrane of the mitochondria. The acyl-carnitine can enter the intermembrane space of the mitochondria. The carnitine-acylcarnitine translocase (CACT) on the inner mitochondrial membrane transports acyl-carnitine into the mitochondrial matrix in exchange for free L-carnitine. In the mitochondrial matrix, CPT2 de-esterifies acyl-carnitine into acyl-CoA and L-carnitine [53]. The oxidation of dietary FAs in NAFLD patients is considerably low compared to healthy subjects. A breath-test study using ^13^C-labeled palmitic acid estimated that FA *β*-oxidation in NAFLD patients is 27% lower than in healthy subjects [54]. This reduction in FA oxidation is attributed to the disruption of FA *β*-oxidation under NAFLD conditions. The reduction in L-carnitine and acyl-carnitine levels in mice with hepatic lipid accumulation signifies the importance of FA *β*-oxidation for hepatic lipid homeostasis [55]. Similar to L-carnitine, the hepatic levels of CPT1 considerably decline in NAFLD, suggesting impaired mitochondrial FA *β*-oxidation [56]. However, disruption of FA oxidation may not occur in early NAFLD but only once the disease progresses to NASH. In early NAFLD, the mitochondria may adapt to the excessive influx of FAs by increasing FA *β*-oxidation. A study comparing liver biopsies revealed that hepatic mitochondrial respiration in obese individuals with or without steatosis is 4.3–5.0 times higher compared to healthy lean individuals. Mitochondrial respiration declines by 31–40% when steatosis progresses into NASH [57]. Peroxisome proliferator-activated receptor (PPAR)-*α* is a master regulator of mitochondrial FA *β*-oxidation. Activation of the PPAR-*α* can promote FA *β*-oxidation in hepatocytes (Figure 2). PPAR-*α* is activated by multiple stimulators, such as postprandial insulinemia, FA ligands derived by lipogenesis and TG hydrolysis, glucagon secreted during fasting, and AMPK-mediated energy generation [58]. Apart from hepatic glucose and lipid metabolism, PPAR-*α* plays a key role in preventing hepatic inflammation through multiple mechanisms. The FA *β*-oxidation promoted by PPAR-*α* prevents the excessive accumulation of hepatic lipids ready to undergo lipid peroxidation and subsequent reactive oxygen species (ROS) generation. Reduction of ROS generation (oxidative stress) can ameliorate hepatic inflammation and fibrosis induced by hepatocyte damage [58]. Moreover, PPAR-*α* can directly inhibit proinflammatory transcription factors, such as NF-*κ*B, activator protein (AP)-1, and signal transducer and activator of transcription (STAT), to mitigate hepatic inflammation [59]. Analysis of the liver biopsies revealed a negative association between the PPAR-*α* expression and the severity of NASH [60]. Thus, activation of PPAR-*α* is believed to be a viable therapeutic target for the mitigation of NAFLD. Reactivation of PPAR-*α* by using fenofibrate (a PPAR-*α* agonist) could significantly reduce liver damage markers and improve lipid metabolism in NAFLD patients. Moreover, PPAR-*α* activation prevented the loss of lysosomal acid lipase (LAL) activity under NAFLD conditions in vitro. LAL prevents hepatic lipid accumulation by the hydrolysis of TG and cholesterol esters to FFA [61]. Disruption of FA *β*-oxidation is a major contributor to the pathogenesis of NAFLD. PPAR-*α* plays a critical role in maintaining hepatic lipid homeostasis by regulating the FA *β*-oxidation.

### 2.3. TG Secretion in VLDL

The hepatic excretion of TG with VLDL can be considerably increased in NAFLD patients [62]. The assembling of VLDL for TG excretion is a two-step process that occurs in the ER. Initially, TG is transferred onto apolipoprotein B (ApoB) by the microsomal triglyceride transfer protein (MTP) to form small and dense VLDL precursors. These VLDL precursors undergo maturation by fusion with the protein-free TG droplets in the ER [63]. Mutations of the ApoB and MTP, such as in familial hypobetalipoproteinemia [64] and abetalipoproteinemia [65], respectively, can increase the risk of NAFLD by impaired TG secretions, such as VLDL. In NAFLD patients, the increased TG secretion in VLDL is evident by the increased blood serum VLDL concentrations and upregulated expression of ApoB100 and MTP [62]. However, the expression of ApoB and MTP are significantly low in the NASH patients compared to the patients with simple hepatic steatosis, suggesting possible impairment of TG secretions such as VLDL with the progression of steatosis into NASH [62]. A study on the VLDL secretion rate in nondiabetic obese subjects revealed that the rate of VLDL secretion depends on the intrahepatic lipid (IHL) content. The VLDL secretion rate increases up to 5% of IHL accumulation, plateaus between 5–10%, and declines thereafter [66].

### 2.4. Adipose Tissue Dysfunction

Obesity-related adipose tissue dysfunction is associated with the pathogenesis of steatosis and progression to NASH (Figure 2). Human adipose tissues can be categorized as visceral and subcutaneous based on the anatomical location, and as white adipose tissue (WAT) and brown adipose tissue (BAT) based on color [67]. Both subcutaneous and visceral fat contribute to the development of NAFLD. However, a recent study suggests that the amount of visceral fat is more closely associated with the severity of NAFLD compared with subcutaneous fat content [68]. Moreover, the density of visceral fat has a higher association with the prevalence of NAFLD compared with the amount (area) of visceral fat [69]. The WAT is primarily specialized for energy (TG) storage, while BAT is for thermogenesis [70]. The unique expression of uncoupling protein 1 in the BAT uncouples the mitochondrial respiration to facilitate the release of energy as heat [67]. Even though BAT can spend dietary FAs as heat, this dietary FA clearance is trivial compared with the heart, liver, skeletal muscles, and WAT. Only about 0.3% of dietary FAs are cleared by BAT in cold-acclimated men [71]. Insulin resistance and inflammation in WAT can significantly increase the risk of NAFLD pathogenesis. Insulin resistance in the adipose tissue can impair glucose uptake [72], increasing the circulating glucose that can induce hepatic TG accumulation by upregulating DNL [73]. This impairment of glucose uptake in insulin resistance is associated with the reduced expression of insulin-regulated glucose transporter type 4 (GLUT4) [72]. Under insulin resistance, the diminished anti-lipolytic activity of insulin causes excessive release of FFA into the bloodstream, which can induce hepatic TG accumulation [74]. The vascular endothelial growth factor B (VEGF-B) can contribute to NAFLD pathogenesis by promoting lipolysis in the WAT. The expression of VEGF-B significantly increases in the WAT under obesity and NAFLD. Also, the expression of VEGF-B positively correlates with the expression of genes upregulating lipolysis in WAT, and FA uptake, DNL, and inflammatory progression of the steatosis in the liver [75].

The PPAR-*γ* is a central regulator of FA metabolism in the adipocytes (Figure 2). Activation of the PPAR-*γ* increases the transport of FFA into the adipocytes by upregulating the expression of CD36, adipocyte protein 2, and LPL. PPAR-*γ* facilitates the conversion of influx FFA into TG for storage by upregulating the expression of the phosphoenolpyruvate carboxykinase (PEPCK) enzyme. PEPCK enzyme is responsible for supplying the glycerol backbone required for the esterification of FFA into TG. Also, in adipose tissue, PPAR-*γ* increases the secretion of adiponectin that can ameliorate NAFLD by promoting hepatic insulin sensitivity and *β*-oxidation, restricting hepatic gluconeogenesis, and suppressing the production of proinflammatory cytokine tumor necrosis factor (TNF)-*α* [76]. The adipocyte expression of PPAR-*γ* may deplete under the conditions favoring NAFLD pathogenesis. A significant reduction in the expression (at mRNA level) and DNA binding ability of the PPAR-*γ* in epidermal WAT had been observed in C57BL/6J mice fed with an HFD [77]. Thus, activation of the PPAR-*γ* in adipose tissues can be beneficial to mitigate the risk of NAFLD. In contrast to the adipose tissue, expression of the PPAR-*γ* is upregulated in the liver under NAFLD. A study evaluating obese–steatosis and obese–NASH patients revealed that the hepatic expression of PPAR-*γ* can increase largely by 112% and 188%, respectively, at the mRNA level [78]. Activation of the PPAR-*γ* in the liver promotes lipid accumulation similar to in the adipocytes [76]. Targeted deletion of the PPAR-*γ* in hepatic tissue could significantly reduce NAFLD pathogenesis in HFD-fed mice by downregulating the expressions of FA transporters (CD36, liver-type FA-binding protein, and MTP) and DNL promoters (stearoyl-CoA desaturase 1, SREBP-1, and ACC). However, the deletion of PPAR-*γ* downregulated the expression of *β*-oxidation promoters PPAR-*α* and acetyl-CoA oxidase in the mice livers [79]. Functions of the PPAR-*γ* in NAFLD pathogenesis are tissue-specific, with underexpression in adipose tissue and overexpression in the hepatic tissue favoring the pathogenesis and progression of the disease.

### 2.5. Steatosis Progression to NASH

Traditionally, the progression of simple steatosis into NASH and fibrosis is described by the two-hit hypothesis. Overnutrition, obesity, insulin resistance, or metabolic syndrome can act as the first-hit to induce hepatic lipid accumulation and lipid peroxidation. The second-hit continues the hepatic assault by introducing hepatocyte injury and inflammation. The second-hit is driven by hepatocellular oxidative stress, mitochondrial dysfunction, Fas ligand activation, and proinflammatory cytokines production. The endotoxins of gut microbiota can assist the second-hit by promoting hepatic inflammation through innate immune responses [80]. However, recent studies suggest that the pathogenesis and progression of NAFLD are driven by multiple parallel hits. The multiple-hits hypothesis describes the possibility of the simultaneous occurrence of several hepatic assaults in the subjects genetically predisposed to NAFLD [81]. Insulin resistance, hepatic ER and oxidative stresses, mitochondrial dysfunction, adipose tissue lipotoxicity, gut microbiota dysbiosis, and genetic predisposition are the major hits recognized in the multiple-hits hypothesis [81,82].

#### 2.5.1. ER Stress in NAFLD Progression

**Figure 3:**
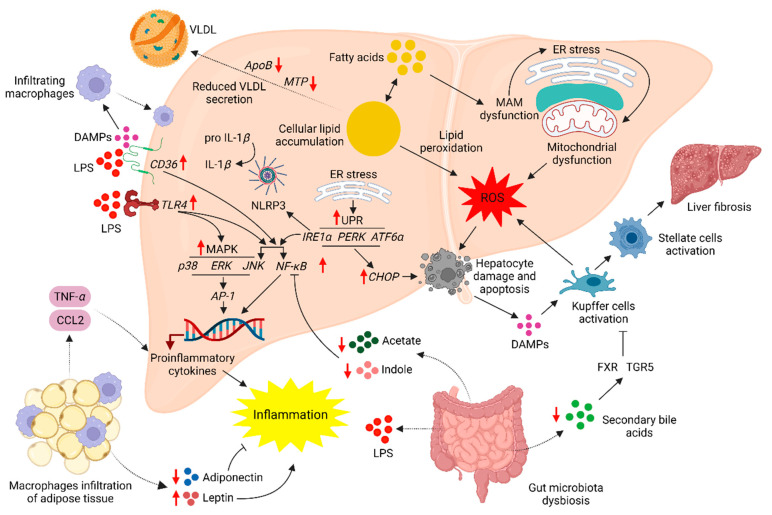
Mechanisms of steatosis progression to nonalcoholic steatohepatitis. The red arrows indicate the increased or decreased expression/production under nonalcoholic steatohepatitis. ApoB, apolipoprotein B; ATF6α, activating transcription factor-6α; CCL2, C-C motif chemokine ligand 2; CD36, cluster of differentiation 36; CHOP, CCAAT-enhancer-binding protein-homologous protein; DAMPs, damage-associated molecular patterns; ER, endoplasmic reticulum; ERK, extracellular signal-regulated kinase; FXR, farnesoid X receptor; IL, interleukin; IRE1α, inositol-requiring enzyme-1α; JNK, c-Jun-N-terminal kinase; LPS, lipopolysaccharide; MAM, mitochondria-associated ER membrane; MAPK, mitogen-activated protein kinase; MTP, microsomal triglyceride transfer protein; NF-κB, nuclear factor-kappa-light-chain-enhancer of activated B cells; NLRP3, nucleotide-binding domain, leucine-rich-containing family, pyrin domain-containing-3 inflammasomes; PERK, protein kinase R-like ER kinase; ROS, reactive oxygen species; TGR5, Takeda G protein-coupled receptor 5; TLR4, toll-like receptor 4; TNF-α, tumor necrosis factor-α; UPR, unfolded protein response; VLDL, very-low density lipoprotein (created with BioRender.com, license purchased, accessed on 14 November 2023).

ER stress assists the progression of steatosis to NASH through multiple mechanisms (Figure 3) associated with the unfolded protein response (UPR) [83]. Several studies have illustrated the positive correlation between hepatic lipid accumulation and ER stress both in vitro [84] and in vivo [85]. The mitochondria-associated ER membranes (MAMs) bridge the ER and mitochondria in hepatocytes both functionally and structurally. Lipotoxicity in hepatocytes can disrupt MAMs both functionally and structurally. A significant reduction in the calcium flux from ER to mitochondria, together with shrinkage of the MAMs contact area, had been observed in the HepG2 cells overloaded with palmitic acid. This disruption of MAMs is assumed to be mediated by the increased distance between the ER and mitochondria through the downregulation of the expression of the MAMs structural component mitofusin-2 [86]. Disruption of the MAM leads to ER and oxidative stresses, promoting the progression of NAFLD [87]. Apart from palmitic acid, free cholesterol, lysophosphatidylcholine, and sphingolipids, such as ceramides, are known activators of hepatic ER stress [88]. ER stress is manifested by the accumulation of unfolded protein in the ER lumen. The UPR attempts to resolve ER stress by activating three ER transmembrane stress sensors (Figure 3), namely, protein kinase R-like ER kinase (PERK), inositol-requiring enzyme-1*α* (IRE1*α*), and activating transcription factor-6*α* (ATF6*α*) [83]. These three stress sensors are capable of activating the intrinsic apoptotic pathway through CCAAT-enhancer-binding protein (C/EBP)-homologous protein (CHOP) signaling [89]. IRE1*α* activation assists NAFLD progression by inducing inflammatory responses through the nucleotide-binding domain, leucine-rich-containing family, pyrin domain-containing-3 (NLRP3) inflammasomes, and NF-*κ*B and JNK signaling [83]. Also, activation of IRE1*α* in the liver resident macrophages, the Kupffer cells, can induce hepatic ischemia/reperfusion (I/R) injury. Inhibition of IRE1*α* in mice induced for I/R injury had depicted significant reductions in the hepatic infiltration of Ly6G^+^ neutrophils, and serum levels of interleukin (IL)-1*β* and TNF-*α* proinflammatory cytokines together with C-C motif chemokine ligand 2 (CCL2) and C-X-C motif chemokine ligand 10 (CXCL10) proinflammatory chemokines [90]. Thus, ER stress is a primary driver of the progression of hepatic steatosis to NASH. Moreover, ER stress may assist in sustaining hepatic lipid accumulation by impairing mitochondrial *β*-oxidation [91].

#### 2.5.2. Oxidative Stress in NAFLD Progression

Cellular ER stress can induce oxidative stress through mitochondrial dysfunction-mediated ROS generation [92]. Also, increased FA *β*-oxidation in the mitochondria and peroxisomes and activation of the nicotinamide adenine dinucleotide phosphate oxidase (NOX) enzyme can induce cellular oxidative stress during NAFLD by promoting the production of ROS [93]. Cellular oxidative stress is a primary modulator of NAFLD pathogenesis and progression (Figure 3). A recent study has proposed that increased cellular oxidative stress can promote cellular lipid accumulation by upregulating DNL via SREBP-1 activation [94]. The ability of ROS to damage cellular protein, lipids, and nucleic acids promotes hepatic apoptosis through both structural and functional damage to the hepatocytes [95]. Liver resident macrophages, the Kupffer cells, engulf apoptotic bodies produced by the hepatocytes undergoing apoptosis. Engulfment of the apoptotic bodies activates the Kupffer cells to produce TNF-*α*-related apoptosis-inducing ligand (TRAIL), Fas, and TNF-*α* cell death ligands capable of further promoting hepatic apoptosis [96]. Hepatic oxidative stress and inflammation are interdependent and occur simultaneously to exacerbate hepatic damage [97]. Increased hepatic oxidative stress promotes hepatic inflammation by the production of proinflammatory cytokines, IL-1*β* and IL-18, through MAPK and NF-*κ*B signaling [98]. Activation of the MAPK and NF-*κ*B inflammatory signaling had also been observed in HepG2 cells overloaded with glucose. Hyperglycemia in HepG2 cells could promote TNF-*α* and IL-6 production through ROS-induced MAPK and NF-*κ*B signaling [99]. Proinflammatory cytokines, such as TNF-*α* [100] and DAMP, can activate the inflammatory responses of the Kupffer cells [101]. DAMP further intensifies hepatic inflammation by recruiting monocyte-derived macrophages (MDM), also termed infiltrating macrophages. Both Kupffer cells and MDM expand the hepatic inflammatory assault by the production of proinflammatory cytokines and chemokines [101]. Once activated, Kupffer cells may promote hepatic oxidative stress through ROS production [102], thus initiating a harmful cycle of oxidative stress and inflammation. Moreover, ROS can activate hepatic stellate cells (HSC) into a myofibroblast-like phenotype and stimulate HSC to produce extracellular matrix (ECM). Excessive ECM production by the HSC leads to the progression of NAFLD into liver fibrosis and cirrhosis [103]. Hepatic oxidative stress accelerates the progression of steatosis to NASH and the subsequent development of hepatic fibrosis and cirrhosis.

#### 2.5.3. Adipose Tissue Dysfunction for NAFLD Progression

Adipose tissue dysfunction may contribute to hepatic inflammation by increasing the levels of proinflammatory cytokines such as TNF-*α* and IL-6 in the circulation (Figure 3). These cytokines primarily originate from the macrophages that infiltrate the adipose tissue in response to adipocyte apoptosis [104]. Adipose tissue dysfunction is positively associated with obesity [105]. Significant increments in the circulating TNF-*α* and CCL2 levels have been observed in obese individuals. The circulating TNF-*α* and CCL2 increments are similar between the obese subjects presented with and without NAFLD [106], suggesting the sustained adipose tissue-mediated inflammation during the pathogenesis and progression of NAFLD. Also, adiponectin and leptin secreted by the adipose tissue assist NAFLD pathogenesis and progression when deviating from normal physiological levels. Other adipokines such as resistin, visfatin, chemerin, retinol-binding protein 4 (RBP-4), and irisin are assumed to be important for regulating hepatic lipid metabolism and inflammation [107]. In NAFLD patients, the serum adiponectin concentration is considerably low, and this hypoadiponectinemia condition may promote the progression of steatosis to NASH [108]. Adiponectin can prevent hepatic injury by restricting monocyte adhesion to endothelial cells and reducing the expressions of TNF-*α* and aldehyde oxidase-1 enzyme. Aldehyde oxidase-1 induces hepatic oxidative stress and liver injury together with fibrosis [109]. In contrast to adiponectin, the serum concentration of leptin is significantly high in NAFLD patients. A study based on US population data revealed that serum leptin concentration is considerably elevated in NAFLD patients, and the leptin concentration is positively associated with the severity of NAFLD [110]. The ability of leptin to promote hepatic inflammation by upregulating the expressions of CCL2 and IL-6 and induce hepatic fibrosis by the activation of HSC has been demonstrated in a cholestasis mouse model [111]. Adipokine dyshomeostasis contributes to the progression of NAFLD by advancing hepatic inflammation and fibrosis.

### 2.6. Gut Microbiota Dysbiosis in NAFLD Pathogenesis and Progression

The gut microbiota plays a pivotal role in regulating body energy metabolism and immune response [112]. Dysbiosis of the gut microbiota is associated with obesity-related diseases, including NAFLD [113]. Studies analyzing fecal material have revealed that the Firmicutes/Bacteroidetes (F/B) ratio is significantly higher in NAFLD patients compared with healthy individuals [114,115]. The gut microbiota diversity is significantly low in NAFLD patients, and the abundance of bacteria phyla Proteobacteria and Fusobacteria is significantly high in NAFLD patients [116]. One study suggests that the increased abundance of Proteobacteria is the most important alteration in the gut microbiota to favor NAFLD pathogenesis through the gut–liver axis. Proteobacteria are Gram-negative bacteria with lipopolysaccharides (LPS) in the outer membrane that are capable of inducing hepatic steatosis and inflammation [117]. The composition of the gut microbiota alters with the progression of NAFLD. The abundance of the *Lachnospiraceae* family and its descendant *Blautia* genus are significantly higher in NASH patients, whereas the abundance of the *Enterobacteriaceae* family and its descendant Shigella genus are higher in fibrosis patients [116]. Depletion of the *Blautia* genus bacteria using antibiotics restricts NASH development in mice fed with a choline-low HFHS diet mimicking the Western diet. The re-establishment of the *Blautia* genus bacteria significantly intensified the hepatic inflammation and fibrosis in these mice [118]. These pathogenic effects are believed to be mediated by the production of 2-oleoylglycerol in the gut through interactions between *Blautia* genus bacteria and the Western diet. 2-Oleoylglycerol produced in the gut can reach the liver through the portal vein and activate the macrophages. The activated macrophages produce transforming growth factor (TGF)-*β*1, which activates the HSC and upregulates the expression of ECM genes to promote hepatic fibrosis [118]. Gut microbiota dysbiosis was also observed in lean NAFLD patients. However, unlike in obesity-mediated NAFLD, the F/B ratio is significantly lower in lean NAFLD patients compared with healthy individuals [119].

Bile acid metabolism by the gut microbiota is important to maintain glucose and lipid homeostases. The gut microbiota converts the conjugated primary bile acids (cholic and chenodeoxycholic acids) into secondary bile acids (deoxycholic and lithocholic acids) through multiple reactions involving deconjugation, oxidation, 7-dehydroxylation, esterification, and desulfation [120]. Bile acids act as detergents to facilitate the absorption of dietary lipids, steroids, and lipid-soluble vitamins. Also, the bile acids can function as signaling molecules to maintain bile acid and body energy homeostases by stimulating the nuclear farnesoid X receptor (FXR) and membrane Takeda G protein-coupled receptor 5 (TGR5) receptors [121]. Activation of the FXR receptor restricts hepatic lipid accumulation through multiple mechanisms (Figure 3). FXR can inhibit TG synthesis by downregulating the expressions of lipogenic FAS and stearoyl CoA desaturase (SCD)-1 enzymes [122] via SREBP-1c inhibition [123]. FXR activates the small heterodimer partner (SHP), which downregulates the SREBP-1c expression by inhibiting the liver X receptor (LXR) [123]. Also, FXR activation may ameliorate hepatic lipid accumulation by promoting FA *β*-oxidation via upregulation of the PPAR-*α* expression [124]. However, a recent study demonstrated that FXR can inhibit hepatic lipogenesis through a SHP and SREBP-1c independent mechanism, and the activation of intestinal cell FXR is sufficient to ameliorate hepatic lipid accumulation by reducing dietary lipid absorption [125]. Moreover, FXR activation in immune cells can manifest anti-inflammatory and antifibrogenic activities. FXR stimulation inhibits the activation of circulating immune cells together with the Kupffer cells and the subsequent activation of the HSC [126]. Similar to the FXR, activation of the TGR5 is important to maintain energy and immune homeostases [127]. Overexpression of the TGR5 in enteroendocrine L cells induces secretion of glucagon-like peptide (GLP)-1 and improves glucose tolerance in obese mice [128]. GLP-1 can promote insulin secretion and pancreatic islet survival and proliferation. Moreover, in mice, TGR5-mediated GLP-1 secretion promotes energy expenditure by the BAT and muscle tissues [128], thus reducing the risk of obesity-related NAFLD. The importance of TGR5 for the suppression of hepatic inflammation had been demonstrated in an LPS-mediated inflammation mouse model. Deletion of the TGR5 in mice significantly increased hepatic inflammation and necrosis. Anti-inflammatory effects of the TGR5 are mediated through the inhibition of NF-*κ*B-based inflammatory response in the mice macrophages, Kupffer cells, and hepatocytes [129]. Even though anti-inflammatory effects are important to prevent liver fibrosis by inhibiting the activation of HSC, a recent study has revealed the unbeneficial potential of TGR5 to promote liver fibrosis. 12*α*-Hydroxylated bile acids, taurodeoxycholate, and glycoldeoxycholate could activate the HSC and induce fibrogenesis in mice by the activation of TGR5-mediated MAPK, extracellular signal-regulated kinase (ERK) 1/2 and p38 [130]. However, many researchers believe that activation of the FXR and TGR5 present therapeutic targets to ameliorate NAFLD. Gut microbiota dysbiosis in NAFLD is associated with the reduced conversion of primary bile acids into secondary bile acids and low activity of bile acid receptors, FXR, TGR5, pregnane X receptor, and vitamin D receptor [120]. Thus, gut microbiota dysbiosis promotes the pathogenesis and progression of NAFLD by disrupting bile acid metabolism.

The metabolic products and cellular components of the gut microbiota can be both beneficial and detrimental in the NAFLD (Figure 3). The gut microbiota is capable of producing short-chain FAs (SCFAs) such as acetate, propionate, and butyrate by fermenting the resistant starches [131]. In a high-fat/fructose/cholesterol-fed mouse model, supplementation with the resistant starch inulin significantly reduced hepatic steatosis and fibrosis. These hepato-protective effects are attributable to the production of acetate by the gut microbiota-mediated fermentation of inulin. The acetate stimulates the FFA receptor 2 (FFAR2), which may reduce the NAFLD pathogenesis and progression by reducing insulin resistance, NF-*κ*B and TNF-*α*-mediated inflammation, and expression of collagen promoters [131]. In NASH and NAFLD-cirrhosis patients, the SCFA concentrations in blood plasma are significantly diminished. Moreover, the negative association between the blood plasma concentrations of SCFA and proinflammatory cytokine TNF-*α* in these patients [132] suggests the importance of SCFA in reducing NAFLD-related inflammation. However, another study contradicts these findings by reporting elevated levels of fecal SCFA and SCFA-producing gut bacteria in NASH patients [133]. Moreover, elevated fecal acetate and propionate levels may be associated with increased inflammation in NASH patients as observed by the diminished anti-inflammatory regulatory T-cells (Tregs) and the increased ratio of proinflammatory T helper 17 cells (Th17)/Tregs in the blood [133]. Thus, further studies are required to understand the functions of gut microbiota-derived SCFA in NAFLD pathogenesis and progression. Indole and indole derivatives produced by the gut microbiota-mediated metabolism of tryptophan have depicted multiple hepatoprotective effects [134]. Oral administration of indole significantly reduced hepatic inflammation in LPS-injected mice by restricting the NF-*κ*B signaling and the expressions of its downstream targets, notably IL-1*β*, IL-6, IL-10, TNF-*α*, nitric oxide synthase 2 (NOS2), and NOX1 [135]. Similarly, anti-inflammatory activity, together with a reduction in hepatic macrophage infiltration, has been observed for indole-3-acetate in mice induced for the NAFLD by an HFD. Also, indole-3-acetate could improve the blood glucose and lipid profiles and ameliorate the hepatic TG and cholesterol content by downregulating the expression of lipogenic SREBP-1, SCD-1, ACC1, and PPAR-*γ*. Moreover, the potential of indole-3-acetate to mitigate HFD-induced oxidative stress is important to prevent the pathogenesis and progression of NAFLD [136]. In NAFLD, hepato-protective effects of the indole and indole derivatives may be diminished by the disruption of tryptophan metabolism due to gut microbiota dysbiosis [137].

Gut microbiota dysbiosis in the NASH can lead to an increased abundance of ethanol-producing bacteria and a higher concentration of endogenous ethanol in the peripheral blood. The gut microbiota of obese individuals and NASH patients are similar. However, the abundance of ethanol-producing *Escherichia* bacteria and blood ethanol concentrations are higher in NASH patients compared to obese individuals [138]. Endogenous ethanol can induce hepatic steatosis and injury in mice by prompting mitochondria dysfunction-mediated oxidative stress [139]. Thus, trivial changes in the gut microbiota composition might be sufficient for the progression of NAFLD. Gut microbiota dysbiosis in the NAFLD leads to the disruption of gut epithelial barrier function. Gut bacteria-derived metabolites such as endogenous ethanol and ethanol metabolites are capable of disrupting the gut barrier by degrading tight-junction (TJ) protein, allowing gut bacteria and bacterial endotoxins (LPS) to translocate into the liver [140]. Overgrowth of the LPS-producing bacteria such as *Enterobacter cloacae* B29, *Escherichia coli* PY102, and *Klebsiella pneumoniae* A7 in NAFLD patients [141] may further intensify the endotoxin-mediated liver assault. Significantly higher concentrations of LPS had been detected in the blood serum and livers of NASH patients compared to healthy individuals. However, hepatic LPS levels between the simple steatosis patients and healthy subjects had been statistically similar [142], suggesting the importance of bacterial endotoxins for the progression of the NALFD. Activation of the NF-*κ*B inflammatory signaling is the most prominent mechanism in LPS-mediated hepatic inflammation. The LPS initially binds with LPS-binding protein (LBP) and subsequently forms a complex with myeloid differentiation factor 2 (MD2) and pattern-recognition receptor cluster of differentiation 14 (CD14) to activate the toll-like receptor 4 (TLR4) [143]. TLR4 is expressed in both parenchymal hepatocytes and nonparenchymal liver cells, including Kupffer cells, HSC, biliary cells, and endothelial cells. Kupffer cells and HSC are the most stimulated by the LPS to induce inflammatory responses [144]. The activated TLR4 initiates the NF-*κ*B signaling that induces the production of many proinflammatory cytokines, including IL-1*α*, IL-1*β*, IL-6, IL-12, TNF-*α*, and granulocyte-macrophage colony-stimulating factor (GM-CSF) [145]. TLR4 is also capable of activating the MAPK (p38, ERK, and JNK) and interferon regulatory factor 3 (IRF3) signaling to induce hepatic inflammation through the increased production of proinflammatory cytokines [143]. The proinflammatory cytokines secreted by the Kupffer cells and infiltrated macrophages can activate the HSC. Activated Kupffer cells can secrete transforming growth factor *β* (TGF*β*), which stimulates the HSC to produce ECM constituents, collagens, and fibronectin. Excessive synthesis of the ECM leads to hepatic fibrosis [146]. Although many of the LPS-mediated effects support the NAFLD progression into NASH and fibrosis, LPS may also play a role in the induction of hepatic steatosis. LPS could induce hepatic steatosis in a disaccharide-rich diet-fed rat model by upregulating the expressions of lipogenic SREBP-1c and FAS [147]. Moreover, in an HFD-fed germ-free mouse model, monoassociation of the germ-free mice with LPS-producing bacteria was required to induce hepatic steatosis and inflammation [141]. However, LPS was able to ameliorate lipid accumulation in the HSC through lipophagy while sensitizing the HSC to TGF*β*-mediated fibrosis response [148]. Thus, LPS may be playing disease-stage-specific roles in NAFLD. The increased LPS production, together with the impaired gut epithelial barrier function under the gut microbiota dysbiosis, are critical triggers for NAFLD pathogenesis and progression. Gut microbiota dysbiosis is strongly associated with dietary habits such as consumption of fructose-rich diets. Excessive consumption of fructose may induce hepatic inflammation and fibrosis by increasing the translocation of gut microbiota-derived LPS into the liver. Fructose can impair gut epithelial barrier function to increase LPS translocation by downregulating the expression of TJ and adherent junction proteins [149]. NAFLD pathogenesis and progression are contributed to by multiple risk factors. Many of the primary risk factors are associated with modifiable lifestyle patterns.

## 3. Probiotic Microbe-Mediated Mechanisms for NAFLD Risk Reduction

The administration of probiotic microbes is proven to be beneficial in many diseases [150,151]. Probiotics can reduce the risk of NAFLD pathogenesis through multiple mechanisms (Table 1). The ability of probiotics to mitigate insulin resistance and T2D is beneficial for the prevention of NAFLD. A mixture of *Lactobacillus* (*L*.) and *Bifidobacterium* (*B*.) probiotic bacteria has been able to ameliorate HFD and streptozotocin-induced diabetes in C57BL/6J mice by improving glucose tolerance and reducing insulin resistance. These beneficial effects are believed to be mediated by the ability of probiotic bacteria to modulate gut microbiota and increase the availability of GLP-1 and peptide tyrosine–tyrosine through SCFA production [152]. Smoking is a non-traditional risk factor of NAFLD that can promote the progression of steatosis to NASH. In cigarette smokers, nicotine accumulated in the intestine can facilitate the formation of ceramides by phosphorylation of sphingomyelin phosphodiesterase 3 (SMPD3) via AMPK*α* signaling [153]. Ceramides are known activators of hepatic ER stress [88]. The gut bacterium *Bacteroides xylanisolvens* is capable of restricting the formation of ceramides by degrading nicotine, thus reducing the risk of NASH. Therefore, *Bacteroides xylanisolvens* can be a promising novel probiotic bacteria to reduce the risk of NASH in cigarette smokers [153].

Many studies have illustrated the potential of probiotic bacteria to reduce the risk of NAFLD by ameliorating overweight and obesity. A study on obese NAFLD patients revealed that supplementation with a mixture of *Lactobacillus* and *Bifidobacterium* probiotic bacteria can significantly reduce the IHL and triglyceride levels together with the body weight and total fat content [154]. Similarly, mixtures of probiotic bacteria can improve the blood lipid profile and reduce the blood concentrations of liver damage markers, alanine aminotransferase (ALT), aspartate aminotransferase (AST), and *γ*-glutamyl transferase (GGT) [155], demonstrating the ability of probiotic bacteria to regulate lipid metabolism and hepatic injury. Probiotic bacteria such as *L*. *plantarum* Q16 (isolated from yogurt) are capable of preventing hepatic steatosis by the regulation of lipid metabolism. *L*. *plantarum* Q16 has been able to reduce hepatic steatosis in an HFD-fed mouse model by restricting DNL through downregulation of the expressions of SREBP-1 targets, SCD-1, ACC, and FAS [156]. Moreover, *L*. *plantarum* Q16 is capable of upregulating the expression of FA *β*-oxidation promoters, CPT-1*α* and PPAR-*α*. The potential of *L*. *plantarum* Q16 to increase the expression of adipose triglyceride lipase (ATGL) and reduce the expression of diacylglycerol acyltransferase 1 (DGAT1) in the liver is further beneficial for NAFLD mitigation by inhibiting the hepatic TG synthesis. ATGL regulates the TG hydrolysis and is important to maintain PPAR-*α*-mediated FA *β*-oxidation in the mitochondria [156]. DGAT1 can promote hepatic steatosis by esterification of exogenous FA [163].

Adipose tissue dysfunction during obesity is a major contributor to the NAFLD pathogenesis and progression to NASH [164]. The probiotic bacteria *Bacillus coagulans* T4 has been able to ameliorate adipose tissue dysfunction in obese mice [157]. Supplementation with the *Bacillus coagulans* T4 significantly reduced the infiltration of macrophages into the adipose tissue and polarization into the proinflammatory M1 phenotype. Moreover, the mRNA expression of endotoxin receptors, TLR2 and TLR4, together with the proinflammatory cytokines IL-6 and TNF-*α*, was significantly diminished by probiotic bacteria supplementation. This inflammation reduction may be attributable to the ability of *Bacillus coagulans* T4 to reduce endotoxemia by promoting gut epithelial barrier function [157]. The adipokines secreted by the adipocytes are important to maintain hepatic lipid and immune homeostasis [107]. *Lactobacillus* probiotic bacteria are capable of restoring the blood serum adipokine levels in obese mice by increasing the adiponectin level and decreasing the leptin level [158]. Adiponectin deficiency and leptin overexpression can promote hepatic steatosis by restricting FA *β*-oxidation and facilitate the steatosis progression to NASH by advancing hepatic inflammation [76,111].

Hepatic ER stress and oxidative stress can initiate the progression of steatosis to NASH by introducing hepatic inflammation and injury. The ability of probiotic bacteria to mitigate the ER stress response (the UPR) has been demonstrated in HepG2 cells induced for steatosis by exposure to oleic acid. Treatment of the HepG2 cells with the cell-free extracts of *L*. *acidophilus* NX2-6 significantly reduced UPR by downregulating the expressions of glucose-regulated protein 58 kD (GRP58), ATF6, IRE1α, X-box binding protein 1 (XBP1), and CHOP [159]. The potential of probiotic-derived cell-free extract to prevent mitochondria dysfunction [159] may have contributed to mitigating ER stress. Prevention of mitochondrial dysfunction, together with lipid peroxidation and ROS generation, significantly diminished oxidative stress in HepG2 cells [159]. A mixture of 20 strains of lactic acid-producing probiotic bacteria was able to mitigate the hepatic inflammation and subsequent NAFLD progression to fibrosis and HCC in a phosphate and tensin homolog (PTEN) gene knockout mouse model [160]. Hepatocyte-specific deletion of the tumor suppressor gene PTEN mimics NAFLD conditions by inducing hepatomegaly, TG accumulation, inflammation, and hepatic injury [165]. Supplementation with the probiotic bacteria significantly reduced the hepatic oxidative stress by restoring the cellular levels of glutathione in the PTEN knockout mice. Also, in the same mouse model, probiotics significantly reduced the expression of proinflammatory cytokines and averted hepatic injury by reducing hepatocyte apoptosis. Moreover, probiotic bacteria hindered the induction of hepatic fibrosis by downregulating the expressions of tissue inhibitors of metalloproteinase-1 (TIMP-1) and *α*-smooth muscle actin (*α*-SMA) [160]. In hepatic fibrosis, TIMP-1 inhibits the matrix metalloproteases (MMP), which can prevent fibrosis by the degradation of ECM [166]. Increased expression of the *α*-SMA in HSC may induce the secretion of ECM component collagen-1 through mechanical signals such as cellular contraction tension [167].

The gut microbiota dysbiosis can support the pathogenesis and progression of NAFLD through multiple mechanisms [168]. Many studies reporting the benefits of probiotics for NAFLD risk reduction have revealed the potential of probiotics to mitigate gut microbiota dysbiosis. The anti-inflammatory activity of probiotics in NAFLD is primarily attributable to the prevention of dysbiosis-mediated endotoxemia and gut-epithelial barrier dysfunction [169]. During NAFLD, loss of gut-epithelial barrier function allows the translocation of bacteria and bacterial endotoxins to the liver [140], inducing inflammation by activating the TLR4 inflammatory signaling cascade [145]. In an HFD-fed mouse model, supplementation with *L*. *acidophilus* probiotic bacteria relieved the gut microbiota dysbiosis by increasing the bacteria richness and diversity [161]. Also, *L*. *acidophilus* could reduce the F/B ratio [161], which is commonly increased in NAFLD patients [108], and reduce the LPS contributing Gram-negative bacteria [161]. The same study demonstrated the ability of *L*. *acidophilus* to prevent LPS translocation by protecting the gut–epithelial barrier function through increased expression of the intestinal epithelial protein, intectin, and the TJ protein, occludin. Reduction of the LPS translocation significantly ameliorated the hepatic inflammation by inhibiting the TLR4/NF-*κ*B-mediated inflammation pathway [161]. The potential of probiotic bacteria to regulate gut microbiota-derived metabolites is beneficial for NAFLD risk reduction. Supplementation with *L*. *acidophilus* significantly reduced the hepatic steatosis and injury by upregulating the bile acid receptor FXR/fibroblast growth factor 15 (FGF15) signaling, suggesting the potential of probiotic bacteria to mitigate NAFLD through the regulation of bile acid metabolism [162]. Encapsulated *L*. *acidophilus*, *B*. *longum*, and *Enterococcus faecalis* probiotic bacteria can ameliorate gut microbiota dysbiosis in obese mice and restore the abundance of SCFA-producing bacteria such as *Olsenella* and *Allobaculum* [170]. SCFA can reduce NAFLD pathogenesis and progression by inhibiting insulin resistance together with hepatic inflammation and fibrosis [131]. Thus, traditional and novel probiotic bacteria are capable of reducing the NAFLD risk through multiple mechanisms.

## 4. PAC-Mediated Mechanisms for NAFLD Risk Reduction

Supplementation with PAC is beneficial in reducing the risk of NAFLD and alleviating the symptoms in NAFLD patients (Table 2). Oral administration of the PAC can mitigate hyperlipidemia in NAFLD patients together with hepatic injury [171]. Many of the PAC-mediated benefits in NAFLD are associated with the potential of PAC to regulate hepatic energy metabolism. PAC can mitigate insulin resistance in HepG2 cells by activating the phosphoinositide 3-kinase (PI3K)/protein kinase B (AKT) pathway. Activation of the PI3K/AKT signaling by PAC is suggested to be achieved by the upregulation of microRNA (miR)-29a, miR-122, and miR-423 [172]. In oleic acid-overloaded HepG2 cells, PAC can reduce the expressions of lipogenic SREBP-1 and ACC enzyme, together with the cholesterol synthesis enzyme 3-hydroxy-3-methylglutaryl coenzyme A (HMG-CoA) reductase. PAC mediates these metabolic functions by the activation of AMPK energy metabolism pathways [173]. The ability of PAC to mitigate NAFLD pathogenesis has been demonstrated in diet-induced obesity and NAFLD murine models. PAC supplementation can significantly reduce hepatic steatosis by reducing the DNL and lipid storage and promoting FA *β*-oxidation. PAC can significantly reduce the expressions of DNL-promoting mediators such as SREBP-1c, ChREBP, and ACC [174]. The potential of PAC to mitigate hepatic lipid accumulation is demonstrated by the downregulated expressions of the PPAR-*γ* [175] and the lipid droplet-associated proteins, fat-specific protein 27 (FSP27), perilipins, and adipophilin [176]. Also, PAC can increase FA *β*-oxidation by upregulating the expressions of PPAR-*α*, CPT-1, and PPAR-*γ* coactivator-1*α* (PGC-1*α*) [174]. Moreover, PAC may reduce lipid accumulation in the hepatocytes by activating the transcription factor EB (TFEB). TFEB is a master regulator of lysosomal biogenesis. Activation of the TFEB induces lipid degradation through the lysosomal pathway [177].

PAC supplementation can be beneficial to prevent the progression of simple steatosis to NASH. ER stress and oxidative stress act as the primary drivers of NAFLD progression. Supplementation with PAC can significantly reduce HFD-mediated hepatic assault by alleviating ER stress [175]. In obese rats, PAC supplementation mitigated the UPR by downregulating the mRNA expressions of ATF6 and CHOP. Moreover, PAC ameliorated liver injury by reducing hepatocyte apoptosis through the upregulation of antiapoptotic B-cell lymphoma 2 (BCL-2) expression. These beneficial effects are believed to be mediated by the activation of the Wnt-3a/*β*-catenin signaling pathway [175]. Cellular ER stress can promote oxidative stress through mitochondria dysfunction [92]. PACs are natural antioxidants that can relieve hepatic oxidative stress by scavenging ROS and superoxide anion radicals [177]. Also, the potential of PACs to increase the genetic expression and activity of the antioxidant enzymes, glutathione peroxidase (GPx), catalase (CAT), and superoxide dismutase (SOD) is beneficial to mitigate the hepatic oxidative stress in the NAFLD [174,177]. The sustained hepatic injury induces the production of proinflammatory cytokines. Multiple studies have demonstrated the ability of PAC to reduce the production of proinflammatory cytokines, such as IL-1*β*, IL-6, and TNF-*α* [175].

The potential of PAC to ameliorate adipose tissue inflammation is beneficial to reduce the risk of NAFLD. In mice fed with an HFD, PAC could significantly reduce the production of the proinflammatory cytokines in the white adipose tissue and prevent adipokine dyshomeostasis. PAC could increase the blood serum level of adiponectin and decrease the level of leptin [180]. Adiponectin is known to mitigate hepatic inflammation, oxidative stress, and fibrosis [109]. Similarly, reduction of the leptin level can mitigate hepatic inflammation and HSC-mediated hepatic fibrosis [111]. The potential of PAC to mitigate NAFLD progression to hepatic fibrosis has been demonstrated in rats induced for liver damage by CCl_4_ administration [181]. The ability of PAC to downregulate the expressions of collagen-1, *α*-SMA, and TIMP-1 in the HSC is beneficial to prevent hepatic fibrosis by inhibiting excessive ECM secretion. PAC may reduce the excessive ECM secretion from the HSC by inhibiting the PI3K/AKT, MAPK, and NF-*κ*B signaling cascades [182].

Gut microbiota dysbiosis plays a critical role in the pathogenesis and progression of NAFLD [113]. PAC can mitigate HFD-induced gut microbiota dysbiosis by reducing the abundance of *Firmicutes* and increasing the abundance of *Bacteroidetes*. Also, PAC increases the production of SCFA, such as propionic and butyric acids, by increasing the abundance of SCFA-producing bacteria [179]. SCFAs are beneficial in reducing hepatic inflammation and fibrosis [131]. Furthermore, PAC administration can activate the FXR signaling cascade by modulating gut microbiota-mediated bile acid metabolism [178]. Activation of FXR signaling inhibits the hepatic DNL by downregulating the expression of SREBP-1c [123]. Moreover, PAC protects the gut–epithelial barrier function during HFD-induced dysbiosis by upregulating the expressions of TJ proteins, zonula occludens-1 (ZO-1), and occludin. Therefore, PAC reduces the translocation of gut microbiota-derived LPS into the bloodstream [180] and the subsequent activation of the LPS-mediated hepatic inflammatory response [145]. The potential of PAC to modulate lipid metabolism, ER stress, oxidative stress, inflammatory response, and gut microbiota is beneficial to mitigate the risk of NAFLD pathogenesis and progression.

## 5. PAC-Based Synbiotics for NAFLD Risk Reduction

Traditionally, a mixture of probiotics and prebiotics with health benefits was considered a synbiotic. Recently, with emerging new knowledge, synbiotics were re-defined as “a mixture of live microorganism(s) and substrate(s) selectively utilized by host microorganisms that confers a health benefit on the host” [33]. Thus, the new definition allows the use of nonconventional microorganisms and substrates to develop synbiotics, given that the health benefits and safety for use in intended hosts are established. Most of the studies testing the efficacy of synbiotics against NAFLD utilized carbohydrates such as inulin and fructooligosaccharides (FOS) as the synbiotic substrates. The ability of inulin and FOS-based synbiotics to ameliorate hepatic injury [183], steatosis, inflammation, and fibrosis [184,185] was demonstrated in NAFLD patients. The recent findings on synbiotics have revealed the potential to utilize polyphenols as synbiotic substrates for NAFLD risk reduction. Administration of a quercetin-based synbiotic of *Akkermansia muciniphila* has shown beneficial effects in an HFD-fed NAFLD rat model. Administration of this synbiotic significantly reduced hepatic steatosis by downregulating the expressions of lipogenic DGAT2 and C/EBP*α* and upregulating the expression of FA *β*-oxidation promoter PPAR-*α*. Moreover, this synbiotic may mitigate hepatic inflammation by suppressing the expression of proinflammatory cytokine IL-6. These beneficial effects are attributable to the potential of quercetin-based synbiotics to modulate gut microbiota and bile acid metabolism [186].

Studies directly evaluating the potential of PAC-based synbiotics to mitigate the risk of NAFLD are limited. A recent study has evaluated the potential of *Saccharomyces cerevisiae* to biotransform PAC into simple metabolites and the potential of biotransformed PAC to mitigate the pathogenesis of steatosis and its progression to NASH by using AML12 mouse hepatocytes in vitro. The biotransformed PAC could significantly reduce the palmitic acid-induced cellular lipid accumulation in AML12 cells by downregulating the expression of FA transporter CD36, suppressing DNL, and promoting PPAR-*α*-mediated FA *β*-oxidation. This study suggested that biotransformed PAC may be potent in reducing the progression of steatosis to NASH by mitigating cellular inflammation by suppressing TLR4/NF-*κ*B and TLR4/MAPK inflammatory signaling [187]. Similarly, a synbiotic based on the PAC-rich grape seed powder (GSP) and lactic acid bacteria isolated from kefir could significantly reduce the NAFLD risk in an HFD-induced obesity mouse model [188,189]. Supplementation with this GSP synbiotic significantly reduced the body weight gain together with the weights of liver and adipose tissues in the mice while ameliorating insulin resistance, glucose intolerance, and plasma lipid profile [188,189]. Even though the administration of the GSP and probiotic bacteria separately could depict similar activities in the mice, the superiority of synbiotic administration was demonstrated by the significantly lower level of liver damage marker AST in the blood plasma of mice [189]. Analysis of the hepatic gene expressions revealed that GSP synbiotics can modulate cellular signaling to mitigate the NAFLD risk. Most notably, the GSP synbiotic downregulated the expressions of INSIG2, SCD-1, and SCD-3 genes that can regulate hepatic lipogenesis. Also, the GSP synbiotic may ameliorate NAFLD progression by downregulating the expressions of genes related to NF-*κ*B and LPS inflammatory signaling and resolving the hepatic oxidative stress [189]. This reduction of hepatic inflammation may be attributable to the potential of GSP synbiotics to prevent endotoxemia by protecting the gut epithelial barrier function. Furthermore, the ability of GSP synbiotics to modulate adipose tissue lipogenesis and inflammatory response together with the propionic acid (a SCFA) production in the mice gut can be beneficial to mitigate the risk of NAFLD [188]. Moreover, the beneficial effects of the GSP synbiotic correlated with the increased richness of the mice gut microbiota and the abundance of *Akkermansia muciniphila* and *Nocardia coeliaca* bacteria [190]. Interestingly, administration of the GSP alone demonstrated similar activities at comparable levels except for the plasma AST concentration [188,189]. Another synbiotic approach using flavan-3-ol-rich green tea powder and *L*. *plantarum* had reported beneficial effects against NAFLD in HFD-fed mice. This synbiotic approach significantly mitigated insulin resistance, liver damage marker ALT, and hepatic TG level [191]. The expressions of lipogenic enzymes SREBP-1c and PPAR-*γ* were also significantly downregulated in the mice. Moreover, the synbiotic supplementation improved adipose tissue function, as depicted by the suppressed adiposity and blood plasma concentration of leptin. Administration of the green tea powder alone depicted similar beneficial activities at comparable levels. However, the synbiotic administration performed better by suppressing the cholesterol synthesis and hepatic inflammation, as depicted by the downregulated mRNA expressions of the HMG-CoA reductase enzyme and proinflammatory cytokine TNF-*α*, respectively [191]. The development of PAC-based synbiotics may present novel opportunities to reduce the NAFLD risk. Supplementation with epigallocatechin gallate and *L*. *fermentum* can significantly increase the expression of nuclear factor erythroid 2-related factor 2 (Nrf2) in hepatocytes [192]. Similarly, activation of the Nrf2 antioxidant pathway has been recently observed in AML12 cells treated with PAC biotransformed by *S*. *cerevisiae* [187]. Activation of the Nrf2 signaling in NAFLD can prevent hepatic injury by resolving oxidative stress [193].

The development of PAC-based synbiotics can be beneficial to further improve the protective effects of PAC against NAFLD. The majority of dietary PACs are oligomers and polymers [194], and their bioavailability is considerably low [195]. Most of the ingested PAC reaches the colon and undergoes biotransformation into valerolactone derivatives and simple phenolic acids by the colonic microbiota [195]. The beneficial effects of PAC on hepatic lipid metabolism and inflammatory markers significantly depend on the biotransformation into simple metabolites by the gut microbiota. In HFD-fed mice, PAC-mediated improvements to the inflammatory markers correlated with the concentration of procyanidin A2 metabolites produced by the gut microbiota [196]. Thus, biotransformation of the oligomeric and polymeric PAC into absorbable metabolites may promote their potential to mitigate the risk of NAFLD. Therefore, the formulation of synbiotics by combining PAC and probiotic bacteria to biotransform PAC into bioavailable and bioactive metabolites can be a viable strategy to increase the bioactivity of PAC against NAFLD. The potential of probiotic bacteria *L*. *rhamnosus* to biotransform cranberry PAC and the antiproliferative effect of resulting metabolites in the HepG2 liver cancer cells had been demonstrated in vitro [197]. Moreover, PAC-based synbiotics may promote the bioactivities of probiotic bacteria against NAFLD, as PAC is capable of modulating the growth and metabolism of probiotic bacteria. Cranberry PAC can increase the growth of probiotic bacteria *L*. *plantarum*. Also, treatment with PAC can modulate the metabolism of *L*. *plantarum* to utilize FOS, a substrate not generally utilized by the *L*. *plantarum* bacteria [198]. PAC-based synbiotics may mitigate the risk of NAFLD by reducing hepatic lipogenesis, inflammation, and oxidative stress while regulating the adipose tissue function, protecting the gut epithelial barrier function, and modulating the gut microbiota. However, further studies are required to boost PAC–probiotic synergistic activities beneficial for NAFLD risk reduction.

## 6. Conclusions and Future Perspectives

Obesity, insulin resistance, T2D, and MetS, combined with physical inactivity, serve as the primary risk factors for NAFLD. The pathogenesis of steatosis is driven by the increased expression of cellular FA transporters and adipose tissue dysfunction, together with impaired FA *β*-oxidation and VLDL secretion. Oxidative stress and ER stress are the primary drivers of steatosis progression to NASH and fibrosis. Gut microbiota dysbiosis plays a detrimental role in the pathogenesis of steatosis and progression to NASH by promoting LPS-producing pathogens and suppressing the growth of beneficial SCFA-producing bacteria. Moreover, the impairment of gut epithelial barrier function during gut microbiota dysbiosis induces the progression of steatosis to NASH by promoting the translocation of LPS into the liver. Probiotic bacteria, especially *Lactobacillus* and *Bifidobacteria*, and PAC are potent in reducing the risk of steatosis by mitigating obesity, insulin resistance, and T2D. The potential of probiotic bacteria and PAC to suppress DNL and adipose tissue dysfunction while promoting FA *β*-oxidation is beneficial to suppress the pathogenesis of steatosis. Both probiotic bacteria and PAC are capable of reducing the risk of steatosis progression to NASH by beneficially modulating gut microbiota, improving gut epithelial barrier function, and reducing hepatic oxidative and ER stresses. Even though further studies are required, current literature suggests that PAC-based synbiotics are superior in NAFLD risk reduction compared with the individual administration of probiotic bacteria or PAC. Identification of probiotic bacteria capable of PAC biotransformation is essential for the successful development of PAC-based synbiotics. Despite PAC being generally recognized as safe for human consumption [199], it can depict antinutritional effects [200] and digestive tract complications that compromise the gut epithelial barrier function in animals [201,202]. Moreover, probiotic bacteria of *Lactobacillus* and *Enterococcus* can become opportunistic pathogens [203,204]. Therefore, future studies evaluating the effectiveness of PAC-based synbiotics in NAFLD risk reduction must assess the safety for human consumption. The development of PAC-based synbiotics can be a novel solution to counter the increasing global burden of NAFLD.

## Figures and Tables

**Table 1:** Probiotic microbe-mediated functions and mechanisms to reduce nonalcoholic fatty liver disease (NAFLD) risk.

Probiotics	Experimental Model	Biological Functions and Mechanisms	Reference
(1) A mixture of *L*. *acidophilus*, *L*. *rhamnosus*, *L*. *paracasei*, *P*. *pentosaceus*, *B*. *lactis*, and *B*. *breve*.	Obese NAFLD patients were supplemented with the probiotic bacteria mixture (1 × 10^9^ cfu/day) for 12 weeks.	Reduce body weight and total fat content. Reduce intrahepatic fat and triglyceride contents.	[154]
(2) A mixture of *Lactobacillus*, *Bifidobacterium*, and *Enterococcus* probiotic bacteria.	NAFLD patients were supplemented with the probiotic bacteria (1 g twice per day) concomitantly with a low-calorie diet and exercise therapy for three months.	Improve blood lipid profile by reducing the total cholesterol, triglyceride, and LDL levels. Reduce the concentration of liver damage markers, ALT, AST, and GGT in the blood.	[155]
(3) *L*. *plantarum Q16.*	Mice induced for NAFLD by feeding a high-fat diet were supplemented with the probiotic bacteria (1 × 10^9^ cfu/day) for 8 weeks.	Reduce hepatic lipogenesis by downregulating the expressions of SREBP-1, SCD-1, ACC, and FAS. Promote hepatic FA oxidation by upregulating the expressions of CPT-1α and PPAR-α. Promote hepatic triglyceride hydrolysis and reduce triglyceride synthesis by up and downregulation of the expressions of ATGL and DGAT1, respectively. Improve gut microbiota composition.	[156]
(4) *Bacillus coagulans* T4.	C57BL/6J mice were fed a high-fat diet for 10 weeks to induce obesity. Then, the mice were supplemented with the probiotic bacteria (1 × 10^9^ cfu/day) for 8 weeks while feeding with a high-fat diet.	Reduce body weight gain and adiposity and improve glucose tolerance. Reduce infiltration of macrophages into the white adipose tissue and polarization into proinflammatory M1 phenotype. Downregulate the expressions of LPS-sensitive TLR2 and TLR4 in adipose tissue.	[157]
		Downregulate the expression of proinflammatory cytokines TNF-α and IL-6 while upregulating anti-inflammatory IL-10. Ameliorate gut microbiota dysbiosis, improve acetate and propionate SCFA production, and improve gut epithelial barrier function.	
(5) *L*. *casei*, *L*. *fermentum*, *L*. *acidophilus*, *L*. *rhamnosus*, and *L*. *paracesei*.	C57BL/6J mice induced for obesity by feeding with a high-fat diet were administered with the different strains of probiotic bacteria separately.	Improve blood lipid profile by reducing the levels of total cholesterol, triglycerides, and LDL while increasing the level of HDL. Increase blood serum level of adiponectin and reduce the level of leptin.	[158]
(6) *L*. *acidophilus* cell-free extract.	HepG2 cells were treated with the cell-free extract of probiotic bacteria for 24 h. Then, the cells were exposed to oleic acid (0.9 mM) for 24 h to induce lipid accumulation.	Mitigate cellular lipid accumulation by downregulating the expressions of SREBP-1c and FAS. Promote FA oxidation by upregulating the expression of CPT-1. Avert cellular ER stress response (unfolded protein response) by downregulating the expressions of GRP58, ATF6, IRE1α, XBP-1, and CHOP. Ameliorate cellular oxidative stress by preventing mitochondria dysfunction and reducing lipid peroxidation and ROS generation. Reduce cellular inflammation by downregulating the NF-κB-mediated IL-1β and IL-6 production.	[159]
(7) A mixture of 20 strains of lactic acid bacteria.	C57BL/6 mice of hepatocyte-specific phosphate and tensin homolog (PTEN) gene knockout were supplemented.	Ameliorate hepatic oxidative stress and restore glutathione levels. Mitigate lobular inflammation and suppress the expression of LPS receptor TLR4 and proinflammatory cytokines IL-1β, TNF-α, and chemokine CCL2. Alleviate hepatic injury by preventing hepatocyte apoptosis. Reduce the induction of fibrosis by downregulating the expressions of TIMP-1 and α-SMA. May prevent NAFLD progression to hepatocellular carcinoma.	[160]
(8) *L*. *acidophilus.*	C57BL/6J mice were fed a high-fat diet for 11 weeks. Then, supplemented with the probiotic bacteria (5 × 10^9^ cfu/day) and the high-fat diet concomitantly for another week.	Reduce body weight, body fat content, and insulin resistance. Alleviate gut microbiota dysbiosis by increasing bacteria richness and diversity. Reduce the Firmicutes/Bacteroidetes ratio and the abundance of endotoxins carrying Gram-negative bacteria. Protect the gut-epithelial barrier function and reduce the TL4/NF-κB-mediated hepatic inflammation by preventing LPS translocation.	[161]
(9) *L*. *acidophilus* tablet.	Sprague Dawley rats were fed a high-fat diet for 6-weeks. Then, the rats were supplemented with the probiotic tablet (312 mg/kg of body weight/day, 1 × 10^7^ cfu/g of tablet) and high-fat diet concomitantly for 8 weeks.	Mitigate hepatic steatosis and injury by the activation of the bile acid receptor FXR/FGF15 signaling pathway. Ameliorate gut microbiota dysbiosis by increasing bacteria diversity and reducing the abundance of pathogenic bacteria.	[162]

ACC, acetyl-CoA carboxylase; ALT, alanine aminotransferase; AST, aspartate aminotransferase; ATF6, activating transcription factor-6; ATGL, adipose triglyceride lipase; *B*., *Bifidobacterium*; cfu, colony-forming units; CHOP, CCAAT-enhancer-binding protein-homologous protein; CPT-1*α*, carnitine-palmitoyl-transferase-1 *α*; DGAT1, diacylglycerol acyltransferase 1; FAS, fatty acid synthase; FGF15, fibroblast growth factor 15; FXR, farnesoid X receptor; GGT, *γ*-glutamyl transferase; GRP58, glucose regulated protein 58 kD; HDL; high-density lipoprotein; IL, interleukin; IRE1*α*, inositol-requiring enzyme-1*α*; *L*., *Lactobacillus*; LDL, low-density lipoprotein; LPS, lipopolysaccharide; NAFLD, nonalcoholic fatty liver disease; NASH, nonalcoholic steatohepatitis; NF-*κ*B, nuclear factor-kappa-light-chain-enhancer of activated B cells; *P*., *Pediococcus*; PPAR-*α*, peroxisome proliferator-activated receptor-*α*; SCD-1, stearoyl-CoA desaturase 1; SREBP-1, sterol regulatory element binding protein-1; TIMP-1, tissue inhibitor of metalloproteinase-1; TLR, toll-like receptor; TNF-*α*, tumor necrosis factor-*α*; XBP-1, X-box binding protein 1; *α*-SMA, *α*-smooth muscle actin.

**Table 2:** Proanthocyanidin-mediated functions and mechanisms to reduce the NAFLD risk.

Experimental Model	Biological Functions and Mechanisms	Reference
(1) Supplementation of NAFLD patients with PAC-rich grape seed extract (200 mg twice per day) for 2 months.	Ameliorate hyperlipidemia by reducing triglyceride, LDL, and cholesterol levels and increasing HDL levels. Reduce the fasting blood sugar level. Mitigate hepatic injury (as depicted by the low ALT and AST levels).	[171]
(2) C57BL/6 mice were supplemented with the cranberry PAC (200 mg/kg bw/day) and an HFHS diet concomitantly for 12 weeks.	Reduce hepatic lipogenesis by downregulating the expression of SERBP-1c, ChREBP, and FAS. Promote hepatic FA oxidation by upregulating the expressions of CPT-1, PPAR-α, and PGC-1α. Mitigate hepatic inflammation by suppressing NF-κB mediated production of proinflammatory TNF-α and COX-2. Alleviate oxidative stress by upregulating the expressions of GPx, SOD, and Nrf2.	[174]
(3) Wistar rats induced for NAFLD by feeding with an HFHF diet for 30 days were supplemented with grape seed PAC (100 mg/kg bw/day) for another 15 days concomitantly with the HFHF diet.	Reduce hepatic steatosis by downregulating the expressions of lipogenic SREBP-1c and the lipid droplet proteins, FSP27, perilipins, and adipophilin. Promote FA oxidation by upregulating the expression of PPAR-α. Reduce cholesterol synthesis by downregulating the expression of the HMG-CoA reductase enzyme.	[176]
(4) Sprague Dawley rats were fed with an HFD for 8 weeks to induce obesity. The mice were supplemented with grape seed PAC (500 mg/kg bw/day) and HFD concomitantly for 4 weeks.	Mitigate hepatic steatosis by downregulating the expression of PPAR-γ. Alleviate ER stress response (the UPR) by downregulating the expressions of ATF6 and CHOP at the mRNA level. Reduces apoptosis-mediated hepatic injury by upregulating the expression of antiapoptotic BCL-2. Alleviate hepatic inflammation by downregulating the expressions of IL-1β and TNF-α.	[175]
(5) HepG2 and L02 liver cells were exposed to a mixture of oleic and palmitic acids (0.2 mM for 24 h) and treated with procyanidin B2 (2.5–10 µg/mL for 24 h). C57BL/6 mice were fed with an HFD for 10 weeks to induce obesity. Then, mice were administered procyanidin B2 (50 and 150 mg/kg bw/day) and fed with the HFD concomitantly for 10 weeks.	Promote hepatic lipid degradation by activating the TFEB-mediated lysosomal pathway. Mitigate hepatic oxidative stress by scavenging ROS and superoxide anion radicals, protecting the mitochondria membrane potential, preventing glutathione depletion, and increasing the activity of GPx, SOD, and CAT antioxidant enzymes. May reduce hepatic steatosis by restoring hepatic TFEB expression and subsequent lipid degradation by the lysosomal pathway. Mitigate hepatic steatosis by reducing the expressions of PPAR-γ, C/EBPα, and SREBP-1c. Alleviate hepatic oxidative stress by increasing the activity of GPx, SOD, and CAT. Mitigate hepatic inflammation by reducing the production of proinflammatory cytokines, IL-6 and TNF-α.	[177]
(6) An LPS-injected mouse model to evaluate intestinal inflammation. C57BL/6 mice were supplemented with grape seed PAC (250 mg/kg bw/day) for 20 days.	Improve the gut microbiota by increasing bacteria richness and diversity. Increase the mRNA expressions of the bile acid receptor FXR and its targets FGF15 and SHP by modulating the gut microbiota-mediated bile acid metabolism.	[178]
(7) C57BL/6 mice were fed with an HFD containing procyanidin B2 (0.2% *w:w*) for 8 weeks.	Increase the activity of hepatic antioxidant enzymes CAT and SOD. Improve gut microbiota by reducing the abundance of Firmicutes and increasing the abundance of Bacteroidetes. Promote the production of SCFA, propionic, and butyric acids by the gut microbiota.	[179]
(8) C56BL/6J mice were fed with an HFD and administered with PAC isolated from bayberry leaves (100 mg/kg bw/day) for 8 weeks.	Mitigate hepatic lipid accumulation by downregulating the expressions of lipogenic SREBP-1c, ACC, and FAS. Increase FA oxidation by upregulating the expressions of PPAR-α and CPT-1. Increase the blood serum concentration of adiponectin and reduce the concentration of leptin. Protect gut-epithelial barrier function in the ileum and colon by upregulating the expressions of tight junction proteins, ZO-1 and occludin. Reduce the level of circulating LPS and the production of proinflammatory cytokines, IL-6 and TNF-α, in the liver and white adipose tissue.	[180]

ACC, acetyl-CoA carboxylase; ALT, alanine aminotransferase; AST, aspartate aminotransferase; ATF6, activating transcription factor-6; BCL-2, B-cell lymphoma 2; bw, body weight; C/EBP*α*, CCAAT-enhancer-binding protein *α*; CAT, catalase; CHOP, C/EBP-homologous protein; ChREBP, carbohydrate response element-binding protein; COX-2, cycloogenase-2; CPT-1, carnitine-palmitoyl-transferase-1; ER, endoplasmic reticulum; FA, fatty acid; FAS, fatty acid synthase; FGF15, fibroblast growth factor 15; FSP27, fat specific protein 27; FXR, farnesoid X receptor; GPx, glutathione peroxidase; HDL, high-density lipoprotein; HFD, high-fat diet; HFHF, high-fat and high-sucrose; HFHS, high-fat and high-sucrose; HMG-CoA, 3-hydroxy-3-methylglutaryl coenzyme A; IL, interleukin; LDL, low-density lipoprotein; LPS, lipopolysaccharide; NAFLD, nonalcoholic fatty liver disease; Nrf2, nuclear factor erythroid 2-related factor 2; PAC, proanthocyanidin; PGC-1*α*, PPAR-*γ* coactivator-1*α*; PPAR, peroxisome proliferator-activated receptor; ROS, reactive oxygen species; SCFA, short-chain fatty acid; SHP, small heterodimer partner; SOD, superoxide dismutase; SREBP-1c, sterol regulatory element binding protein-1c; TFEB, transcription factor EB; TNF-*α*, tumor necrosis factor-*α*; UPR, unfolded protein response; ZO-1, zonula occludens-1.

## Data Availability

No new data were created or analyzed in this study. Data sharing is not applicable to this article.

## Abbreviations

ACC: acetyl-CoA carboxylase; DNL, de novo lipogenesis; ER, endoplasmic reticulum; FA, fatty acid; FAS, fatty acid synthase; FFA, free fatty acid; HFD, high-fat diet; LCFA, long-chain fatty acid; NAFLD, nonalcoholic fatty liver disease; NASH, nonalcoholic steatohepatitis; PAC, proanthocyanidin; ROS, reactive oxygen species; SREBP-1, sterol regulatory element-binding protein-1; TG, triglycerides; VLDL, very low-density lipoprotein.
